# ACCELERATED CELL DEATH 6 Acts on Natural Leaf Senescence and Nitrogen Fluxes in *Arabidopsis*

**DOI:** 10.3389/fpls.2020.611170

**Published:** 2021-01-07

**Authors:** Sophie Jasinski, Isabelle Fabrissin, Amandine Masson, Anne Marmagne, Alain Lécureuil, Laurence Bill, Fabien Chardon

**Affiliations:** Institut Jean-Pierre Bourgin, INRAE, AgroParisTech, Université Paris-Saclay, Versailles, France

**Keywords:** aging, nitrogen remobilization, nitrogen uptake, seed filling, *Arabidopsis thaliana*, quantitative trait loci, natural variation

## Abstract

As the last step of leaf development, senescence is a molecular process involving cell death mechanism. Leaf senescence is trigged by both internal age-dependent factors and environmental stresses. It must be tightly regulated for the plant to adopt a proper response to environmental variation and to allow the plant to recycle nutrients stored in senescing organs. However, little is known about factors that regulate both nutrients fluxes and plant senescence. Taking advantage of variation for natural leaf senescence between *Arabidopsis thaliana* accessions, *Col-0* and *Ct-1*, we did a fine mapping of a quantitative trait loci for leaf senescence and identified *ACCELERATED CELL DEATH 6* (*ACD6*) as the causal gene. Using two near-isogeneic lines, differing solely around the *ACD6* locus, we showed that *ACD6* regulates rosette growth, leaf chlorophyll content, as well as leaf nitrogen and carbon percentages. To unravel the role of *ACD6* in N remobilization, the two isogenic lines and *acd6* mutant were grown and labeled with ^15^N at the vegetative stage in order to determine ^15^N partitioning between plant organs at harvest. Results showed that N remobilization efficiency was significantly lower in all the genotypes with lower *ACD6* activity irrespective of plant growth and productivity. Measurement of N uptake at vegetative and reproductive stages revealed that *ACD6* did not modify N uptake efficiency but enhanced nitrogen translocation from root to silique. In this study, we have evidenced a new role of ACD6 in regulating both sequential and monocarpic senescences and disrupting the balance between N remobilization and N uptake that is required for a good seed filling.

## Introduction

Leaf senescence constitutes the final stage of leaf development. The most obvious visible symptoms of leaf senescence is yellowing due to chlorophyll degradation. However, during this last developmental stage, other macromolecules are degraded, including macromolecules that have been accumulated through carbon fixation during the photosynthetic period of the leaf. The salvaged nutrient of dying leaf tissues may be remobilized to newly developing organs such as younger leaves, flowers, and seeds (Himelblau and Amasino, 2001). Hence, senescence, although deteriorative by nature, contributes to the growth, reproductive success, and general fitness of plants. Consequently, the onset, rate, and progression of leaf senescence must be tightly regulated to ensure plant survival via the efficient recycling of nutrients for the next generation (i.e., seeds), especially in annual plants.

Leaf senescence is primarily driven by the developmental age but is also regulated by a complex network of internal and environmental signals that are integrated into the age information through intricate regulatory pathways. All phytohormones known to date play a role in leaf senescence regulation. Cytokinins, giberrelins, and auxins delay leaf senescence, whereas ethylene, jasmonic acids, abscisic acid, salicylic acid (SA), brassinosteroids, and strigolactones induce leaf senescence (Jibran et al., 2013; Schippers et al., 2015; Yamada and Umehara, 2015). Other important determinants of leaf senescence are sugar sensing and signaling (Wingler, 2018), as well as the communication between source and sink, corresponding to the demand for nutrients in the sink tissue and the capacity of a source to provide these nutrients (Schippers et al., 2015; Kumar et al., 2019). This source–sink communication is required to adjust the remobilization rate of nutrients. Last but not least, the environment plays a major role in leaf senescence regulation. Darkness, shade, temperature, soil salinity, drought, nutrient limitation, and pathogen infection can all affect senescence for instance (Lim et al., 2007; Schippers et al., 2015; Santos Matos, 2020).

Over the past decade, in an attempt to understand the complex process of leaf senescence, many genetic and molecular studies, together recently with “omics” studies, have been performed in plants, allowing the identification of key regulators as well as intertwined networks involved in leaf senescence regulation (Lim et al., 2007; Guiboileau et al., 2010; Schippers et al., 2015). Multiple layers of regulation, including chromatin, transcriptional, posttranscriptional, translational, and posttranslational regulations, controlled the leaf senescence process (Kim et al., 2018; Woo et al., 2019). All these studies allowed the identification of many transcription factors and gene-regulatory networks. Yet, how these gene networks are coordinated and how this coordination impacts plant fitness and then adaptation of plants to their environment remain poorly understood.

The study of natural variation is a strategy of choice to unravel the role of a trait in plant adaptation and evolution. With this aim, studies have been conducted in *Arabidopsis*, highlighting strong variation in the onset, progression, and intensity of senescence in accessions from different geographic origins (Levey and Wingler, 2005; Luquez et al., 2006; Balazadeh et al., 2008). The genetic basis of this variation was investigated in *Arabidopsis* using recombinant inbred line (RIL) or genome-wide association (GWA) populations for quantitative trait loci (QTL) analysis (Diaz et al., 2006; Luquez et al., 2006; Masclaux-Daubresse et al., 2007; Wingler et al., 2010; Chardon et al., 2014; Lyu et al., 2019). Similar strategies based on the natural variation were carried out to study leaf senescence in various crops such as rice, wheat, sorghum maize, and barley (Wehner et al., 2015; Kamal et al., 2019; Sekhon et al., 2019; Zhao et al., 2019). Recently, the investigation of 259 natural *Arabidopsis* accessions in a GWA study allowed the identification of a new leaf senescence regulator, *Genetic Variants in Leaf Senescence 1* (*GVS1*; Lyu et al., 2019). Interestingly, *GVS1* is also involved in sensitivity to oxidative stress (Lyu et al., 2019), suggesting a link between leaf senescence and oxidative stress. In nature, plants are challenged by many biotic and abiotic stresses, which generate reactive oxygen species and consequently oxidative stress damages. Many studies have previously shown significant overlap, at the molecular level, between senescence and plant defense regulatory pathways (Guo and Gan, 2012). In the same vein, the phytohormones mentioned above regarding leaf senescence regulation are also key players in plant stress responses. This highlights the existence of a crosstalk between senescence and oxidative stress.

During senescence, nitrogen remobilization will allow the seeds to be filled with proteins, which also relies on nitrogen uptake. Similarly to senescence, nitrogen remobilization and uptake are both regulated by genetic and environmental factors (Diaz et al., 2008; Chardon et al., 2010; Masclaux-Daubresse and Chardon, 2011). However, how leaf senescence and nitrogen fluxes are related to each other, in particular to ensure correct seed filling with proteins, is unknown. The links between leaf senescence, yield, and seed filling have been investigated in three *Arabidopsis*-RIL populations, revealing that leaf senescence is negatively correlated to final rosette weight, yield, and seed nitrogen content in the *Ct-1* × *Col-0* population (Chardon et al., 2014). In this population, early senescence decreased the nitrogen remobilization efficiency from the rosette to the reproductive organs and altered seed nitrogen content.

In order to better understand the link between leaf senescence and nitrogen fluxes in the *Ct-1* × *Col-0* population, we aimed to identify the gene underlying the major leaf senescence QTL mapped in this population and to explore its role in nitrogen fluxes. With this objective, we fine mapped the QTL to a single gene, named *ACCELERATED CELL DEATH 6* (*ACD6*), and studied its impact on leaf-aging senescence and its capacity to modulate N mobilization and N uptake during seed filling.

## Materials and Methods

### Plant Material and Growth Condition

The *acd6-2* (SALK_045869, N545869) and *acl1-1* (GK-108H02, N410358) mutants were ordered from the NASC. Both T-DNA mutants were genotyped with gene-specific primers (Supplementary Table S1) flanking the T-DNA insertion site and the T-DNA-specific primers LB-Salk2 (5′-GCTTTCTTCCCTTCCTTTCTC-3′) or gabi_o8409 (5′-ATATTGACCATCATACTCATTGC-3′) for Salk or Gabi mutant, respectively.

**FIGURE 1:**
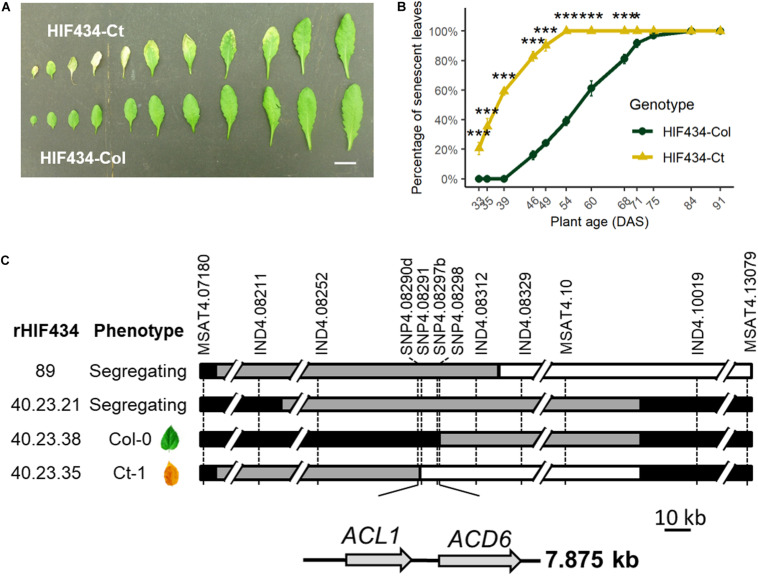
Fine mapping of *SEN.4* locus. **(A)** Rosette leaves (rank 4–14) of 7-week-old plants. Upper row: *HIF434-Ct*; lower row: *HIF434-Col*. Scale bar corresponds to 2 cm. **(B)** Percentage of senescent leaves in *HIF434-Ct* (yellow triangle line) and *HIF434-Col* (dark green circle line) during the reproductive phase. Flowering transition occurred on average at 23.6 days after sowing (DAS) for *HIF434-Col* and 24.6 DAS for *HIF434-Ct*. Stars indicate significant difference between the two genotypes (Student’s test, *n* = 12, *p < 0.05, ***p < 0.001). **(C)** The genotype of the most informative recombinants (rHIFs) used to delineate the final 7.875 kb candidate interval is represented with horizontal bars (black for *Col-0* allele, white for *Ct-1* allele, gray for heterozygous). Dashed vertical bars represent markers used for genotyping between 7.180 and 13.08 Mb on chromosome 4. The phenotype of each rHIF progeny is indicated (phenotype).

The HIF434 was developed from the F8 RIL434 that still segregates in a 5.9-Mb genomic region on chromosome 4 (Tuinstra et al., 1997; Loudet et al., 2005). Several plants were sown and genotyped individually for the appropriate markers across the segregating region, and three independently fixed plants for each allele (named *HIF434-Ct* and *HIF434-Col*, composing the HIF) were chosen and allowed to self-fertilize. In order to identify recombinants (rHIFs) within the segregating interval, 276 F9 plants were genotypically screened. Seventy-seven recombinants were identified and genotyped with microsatellites or indel markers to identify recombination events within the candidate region. Once recombinants had been identified, microsatellites, indel, or dCAPS markers were used to refine and localize recombination breakpoints to smaller intervals when needed. All the markers used are listed in Supplementary Table S2. Twenty-three rHIFs were then tested for the segregation of the leaf senescence phenotype by progeny of fixed-progeny testing. For fixed-progeny testing, for each rHIF, 24 plants were grown and genotyped to isolate individuals fixed for the parental alleles at the remaining heterozygous interval. Three plants fixed for each parental allele were then self-fertilized, and their seed were sawn (four replicates/line) for leaf senescence phenotyping. For progeny testing, for each rHIF, 48 plants were grown and phenotyped for leaf senescence as well as genotyped within the heterozygous interval. The advanced rHIF line 434 (*arHIF434*), which segregates solely for the 7.875-kb candidate region, was obtained from a cross between two different rHIFs lines (rHIF434.40.23.38 and rHIF434.40.23.35; Figure 1C) with adequate genotypes (rHIFs recombined immediately to the north or immediately to the south of the *SEN.4* final interval and with adequate genotype elsewhere), as described by Loudet et al. (2005).

For leaf senescence, plants were grown on soil in greenhouse under natural light supplemented with sodium lamps to provide a 16-h photoperiod. For ^15^N labeling experiments, plant were grown on sand in a growth chamber in short-day condition (8 h light at 140 μmol photons m^–2^ s^–1^, 21 and 17°C day/night temperatures; relative humidity of 65%) until bolting and then transferred to the growth chamber under long-day conditions (16 h light).

### RT-PCR

Three plants per genotype (*Col-0*, *acl1-1*, and *acd6-2*) were grown in greenhouse under long-day condition. At 4 weeks old, the fourth rosette leaves were pooled for total RNA extraction using the RNeasy Plant Mini Kit (Qiagen) following the manufacturer’s protocol. DNAse treatment was performed on columns. Five hundred nanograms of total RNA was reverse transcribed by the RevertAid M-MuLV Reverse Transcriptase (Fermentas) with an oligo (dT) primer according to the manufacturer’s protocol. Complementary DNA (cDNA) was diluted five times, and 2.5 μl was used as template in a 20-μl PCR reaction. PCR primers specific for *ACD6* (ACD6-F1 and SeqACD6-R5), *ACL1* (ACL1-For1 and ACL1-Rev1), and *ACTIN* (ActQ1F and ActQ2R) were used. All sequence primers are described in Supplementary Table S1.

### qRT-PCR

Three plants per genotype (*Col-0*, *acl1-1*, and *acd6-2*) in two independent cultures were grown in greenhouse under long day condition. At 5 weeks old, the sixth rosette leaves were pooled for total RNA extraction using the RNeasy Plant Mini Kit (Qiagen) following the manufacturer’s protocol. DNAse treatment was performed on columns. Two hundred fifty nano grams of total RNA was reverse transcribed by the RevertAid M-MuLV Reverse Transcriptase (Fermentas) with an oligo (dT) primer according to the manufacturer’s protocol. For the qRT-PCR, the 10-μl reaction mixture contained 2.5 μl of cDNA, 0.3 μl of each primer (10 μM), 5 μl of a Takyon Rox SYBR MasterMix dTT Blue solution (UF-RSMT-B0710, Eurogentec, Liège, Belgium) containing the Taq polymerase, the deoxyribonucleotide triphosphates (dNTPs), and the Sybr Green in a reaction buffer, and 2.2 μl of water. The reverse transcription quantitative PCRs (RT-qPCRs) were run on a CFX 96 thermocycler (Biorad) using a first step at 95°C for 3 min and then 40 cycles of 10 s at 95°C, 10 s at 58°C, and 30 s at 72°C. A final step consisted in an increase of 0.1°C s^–1^ to 95°C. The primers used for RT-qPCR are listed in Supplementary Table S1. All primers presented an efficiency of 100 ± 5%. *PP2AA3* (AT1G13320) and *APC2* (AT2G04660) were used as reference genes for the calculation of *ACD6* relative expression.

### Leaf Senescence Phenotyping

Leaf senescence was scored at different time points during plant growth as the ratio of the number of yellow rosette leaves on total number of rosette leaves at bolting.

### Leaf Chlorophyll, Nitrogen, and Carbon Percentage Measurement

Sampling and measurements were done at the same time of the day to avoid circadian effects. The leaves emerging after the cotyledons were numbered continuously from old to young, starting at the two first leaves and ending before the emergence of the cauline leaves, which are recognizable by their small and pointed leaf blade and lack of petioles (Steynen et al., 2001). Chlorophyll content was determined using a Dualex Scientific^TM^ clamp (Force A, Orsay, France). Measurements were taken in the middle of each leaf blade. For each time point during the lifespan of plants, four rosettes for each genotype were harvested. For N and C percentage measurements, leaves were gathered and ground in powder after drying, by groups of 10 leaves: old leaves (OL), ranks 1–10; mature leaves (ML), ranks 11–20; young leaves (YL), ranks 21–30; and new leaves (NL), ranks 31 to >40. A subsample of 1,000–2,000 μg was carefully weighed in tin capsules to determine total C and N percentages of samples using an elemental analyzer (FLASH 2000 Organic Elemental Analyzer, Thermo Fisher Scientific, Courtabeuf, France).

### ^15^N Labeling for Uptake Experiment

Seeds were sown in sand and watered with a 10-mM nitrate solution. Plants were grown in the growth chamber in short days (16 h light, 21 and 17°C day/night temperatures). The vegetative ^15^N uptake time point occurred 40 days after sowing (DAS) when plants were still vegetative. Plants for the postflowering time ^15^N uptake were transferred in long days (16 h light, 21 and 17°C day/night temperatures). The postflowering ^15^N uptake time point occurred 72 DAS, 2 weeks after flower buds emergence. At the time point, the unlabeled watering solution was replaced by an ^15^N-containing solution (10% enrichment). All the pots were watered during 24 h, using an equal volume of labeled solutions. Cutting the rosettes stopped ^15^N uptake. Roots, rosettes, and siliques were then dried, and their dry weight (DW) was determined.

### ^15^N Labeling for Remobilization Experiment

Seeds were sown in sand and watered with a 10-mM nitrate solution. Plants were grown in the growth chamber in short days (8 h light, 21 and 17°C day/night temperatures). Around 40 days after sowing (about 1 week before bolting), 1 ml of a 10-mM nitrate solution containing 10% of ^15^N NO_3_ was dropped to the sand closed to the rosette. After 24 h, plants were rinsed in clear water to eliminate the remaining ^15^N NO_3_. About 10 days after ^15^N labeling, plants were transferred into long-day condition (16 h light, 21 and 17°C day/night temperatures). Plants were harvested at the end of their cycle, at maturity, when all seeds were matured and the rosette dried. Samples were separated as (i) rosette, (ii) stem (stem + cauline leaves + empty-dry siliques), and (iii) seeds (total seeds). The roots were not harvested because a large part of the root system was lost in the sand during harvesting. The DW of rosette, stem, and seeds were determined.

### Determination of ^15^N Abundance

For all the experiments, unlabeled samples were harvested in order to determine the ^15^N natural abundance. After drying and weighting each plant, the material was ground to obtain a homogeneous fine powder. A subsample of 1,000–2,000 μg was carefully weighed in tin capsules to determine the total C and N percentages and ^15^N abundance using an elemental analyzer (FLASH 2000 Organic Elemental Analyzer, Thermo Fisher Scientific, Courtabeuf, France) coupled to an isotope ratio mass spectrometer (delta V isotope ratio mass spectrometer, Thermo Fisher Scientific, Courtabeuf, France) calibrated using international reference (caffeine, IAEA-600, Vienna, Austria). The ^15^N abundance was calculated as atom percent and defined as A% = 100 × (^15^N)/(^15^N + ^14^N) for labeled plant samples and for unlabeled plant controls (A%_*control*_ was ca. 0.3660). The ^15^N enrichment (E%) of the plant material was then defined as (A%_*sample*_ - A%_*control*_)/100. The absolute quantity of N and ^15^N contained in the sample were defined as QtyN = DW × N% and Qty^15^N = DW × E% × N%, respectively. Different parameters used to evaluate harvest index (HI), N fluxes components were defined as follows:

HI=DWseeds/(DWrosette+DWStem+DWseeds),

Nallocationinrosette=QtyNrosette/(QtyNrosette+QtyNstem+QtyNseeds),

Nallocationinstem=QtyNstem/(QtyNrosette+QtyNstem+QtyNseeds),

Nallocationinseeds(NHI)=QtyNseeds/(QtyNrosette+QtyNstem+QtyNseeds),

N15allocationinrosette=QtyNrosette15/(QtyNrosette15+QtyNstem15+QtyNseeds15),

N15allocationinstem=QtyNstem15/(QtyNrosette15+QtyNstem15+QtyNseeds15),

N15allocationinseeds(NHI15)=QtyNSeeds15/(QtyNrosette15+QtyNstem15+QtyNseeds15),

NRE=NHI15/HI,

NUpE=[(Qty⁢Nrosette15+Qty⁢Nstem15+Qty⁢Nseeds15)/E%]/(DWrosette+DWStem+DWseeds)

### Statistical Analyses

Analysis of variance followed by Tukey’s honestly significant difference (HSD) test as well as two−sample t tests were used in this study. All statistical analyses were performed using the free software environment R Version 4.0.2.^1^ The least-square means were calculated using the R package emmeans.

## Results

### *ACD6* Is the Gene Underling the *SEN.4* Leaf Senescence QTL

In a previous study, five QTLs for leaf senescence were mapped in the *Arabidopsis Ct-1* × *Col-0* population (Chardon et al., 2014). The parental accessions were highly contrasted for leaf senescence, *Ct-1* displaying earlier leaf senescence than *Col-0*. In order to gain insight into the leaf senescence molecular process, the QTLs on chromosome 4 (*Ct_Senes_4* and referred as *SEN.4* hereafter), explaining the most important variation (29%), were fine mapped. The *Ct-1* allele displayed an earlier leaf senescence than the *Col-0* allele at *SEN.4*.

The phenotypic effect linked to *SEN.4* was confirmed using specific near-isogenic lines differing for a small genomic region spanning a few megabases around the QTL. Near-isogenic lines for this QTL were obtained by producing a heterogeneous inbred family (HIF), which is easily generated taking advantage of the residual heterozygosity still segregating in RILs (Tuinstra et al., 1997; Loudet et al., 2005). RIL434, segregating only around *SEN.4* but fixed as homozygous for all the tested markers in the rest of chromosome 4 and elsewhere in the genome, was used to generate HIF434. Plants bearing the *Ct-1* allele (“*HIF434-Ct*”) displayed an earlier senescence than plants bearing the *Col-0* allele (“*HIF434-Col*”) (Figures 1A, B), validating the QTL location.

*HIF434* was further used for fine mapping *SEN.4*. Using additional genetic markers, the heterozygous region of *HIF434* was mapped to a 5.9-Mb interval between markers at positions 7.180000 and 13.079020 Mb on chromosome 4. Screening of 276 progeny plants from a *HIF434-Het* individual (heterozygous over the 5.9 Mb region) resulted in the isolation of 77 recombination events in this interval. Phenotyping of the progeny of 24 recombinants (rHIF, see section “Materials and Methods”) reduced the region of interest to a 117.5-kb interval between markers at positions 8.211624 and 8.329176 Mb. A second screening of 1,288 plants resulted in the isolation of 34 new recombinants. Phenotyping of 10 of them reduced the region of interest to a 7.875-kb interval between markers at positions 8.290453 and 8.298328 Mb on chromosome 4 (Figure 1C).

To further confirm this result, an “advanced rHIF cross” (arHIF; see section “Materials and Methods” and Loudet et al., 2008) was designed to obtain the *arHIF434* line, which segregated only for this 7.875 kb region (Supplementary Figure 1A). Like the original HIF, the progeny of this line segregated for leaf senescence with *arHIF434-Ct* displaying an earlier leaf senescence compared to *arHIF434-Col*, confirming the presence of *SEN.4* within this 7.875-kb interval (Supplementary Figures 1B,C).

**FIGURE 2:**
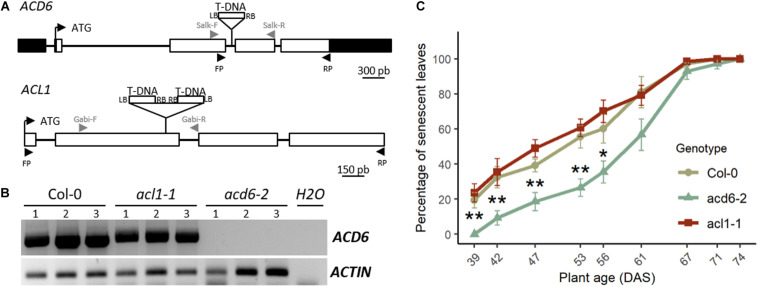
*acd6-2* mutant displays a delayed leaf senescence. **(A)** Structure of the *ACL1* and *ACD6* genes with positions of the T-DNA insertions in *acl1-1* and *acd6-2* mutants is indicated. White boxes represent exons, lines indicate intron, and black boxes represent 5′ and 3′ untranslated regions (UTRs). LB, left border of T-DNA; RB, right border of T-DNA. Gray arrowheads correspond to primers used to genotype mutants. Black arrowheads correspond to primers used for RT-PCR. **(B)** Reverse transcription PCR (RT-PCR) analysis of *ACD6* expression in the fourth leaf of 4-week-old plants of wild type (*Col-0*), *acl1-1*, and *acd6-2*. For each genotype, three different plants (1, 2, and 3) were analyzed. *ACTIN* was used as a constitutively expressed gene control. Primers used for RT-PCR are indicated by black arrowheads in panel **(A)**. **(C)** Percentage of senescent leaves in *Col-0* (khaki round line), *acd6-2* (green triangle line), and *acl1-1* (burgundy square line). Stars indicate significant difference between *Col-0* and *acd6-2* (Student’s test, 7 ≤ *n* ≤ 8, *p < 0.05, **p < 0.01).

Two predicted genes, *ACD6* (*At4g14400*) and *ACL1* encoding an *ACD6*-like ankyrin repeat family protein (*At4g14390*), are present in this 7.875-kb interval (Figure 1C and Supplementary Figure 1A). To investigate the possible role of these two genes in leaf senescence variation between *Ct-1* and *Col-0* accessions, T-DNA insertion mutants in *ACL1* (named *acl1-1*) and *ACD6* (*acd6-2*), both available in the *Col-0* genetic background, were analyzed. Molecular characterization of the mutants revealed that *acl1-1* contained an inverted tandem insertion at the 739th base of the second exon (accompanied with a 54-bp deletion) and *acd6-2* carried a T-DNA insertion in the third intron of *ACD6* (Figure 2A).

Reverse transcription PCR (RT-PCR) using primers specific to *ACD6* (Supplementary Table S1) showed that there is no *ACD6* expression in leaves of *acd6-2* mutant, whereas *ACD6* is strongly expressed in Col-0 and *acl1-1* mutant at the same developmental stage (Figure 2B). Using primers specific to *ACL1* (Supplementary Table S1), no expression of the gene was detected in *Col-0* leaves by RT-PCR, in accordance with an extremely low expression level in rosette leaves as referred in eFP Browser (Winter et al., 2007).

Phenotypic analysis for leaf senescence revealed that *acl1-1* homozygous mutant displayed a leaf senescence kinetic similar to the wild type during plant development (Figure 2C), validating that *ACL1* is not involved in the leaf senescence phenotype. By contrast, *acd6-2* homozygous mutants displayed a delayed leaf senescence compared to wild type (Figure 2C), demonstrating that modification in *ACD6* is responsible for the leaf senescence variation observed at *SEN.4* locus.

Alignment of *ACD6* coding sequences from both *Ct-1* and *Col-0* accessions showed 34 single-nucleotide polymorphisms (SNPs) leading to 20 amino acid changes between both accessions and the lack of the last amino acid in *Ct-1* compared to *Col-0* (Supplementary Figure 2). Two amino acids changes were located in the second ankyrin motif and one was in the eighth one. Five amino acids changes were in transmembrane domains. *Ct-1* and *Col-0* differed at amino acids 566 and 634, showed to be both necessary and sufficient for variation in late-onset leaf necrosis between accessions carrying *ACD6-Est-1* and *ACD6-Col-0* alleles (Todesco et al., 2010). In our study, *Ct-1* displayed the *ACD6-Est-1* allele and Col-0 the *ACD6-Col-0* allele described by Todesco et al. (2010). In addition, we did not detect any variation in *ACD6* transcript levels in the sixth rosette leaf between the two *arHIF434*, at the same stage of development (Supplementary Figure 3A). However, *arHIF434-Ct* plants had higher levels of *SAG12* messenger RNA (mRNA) and lower levels of *RBSC1A* mRNA, confirming that the senescence process was enhanced in *arHIF434-Ct* leaves compared to the *arHIF434-Col* ones (Supplementary Figures 3B,C). The *arHIF434-Ct* plants showed a higher relative expression of *PR1* than *arHIF434-Col* plants, indicating an enhancement of SA signaling in *arHIF434-Ct* leaves (Supplementary Figure 3D). These results suggested that *ACD6* expression was not the source of the early senescence in *arHIF434-Ct*, and they supported that the two modifications at amino acid 566 and 634 were responsible for the leaf senescence variation observed between plants carrying either the *Ct-1* or the *Col-0 ACD6* alleles, although a role of other amino acids cannot be ruled out.

### ACD6 Activity Regulates Leaf Senescence Kinetics

**FIGURE 3:**
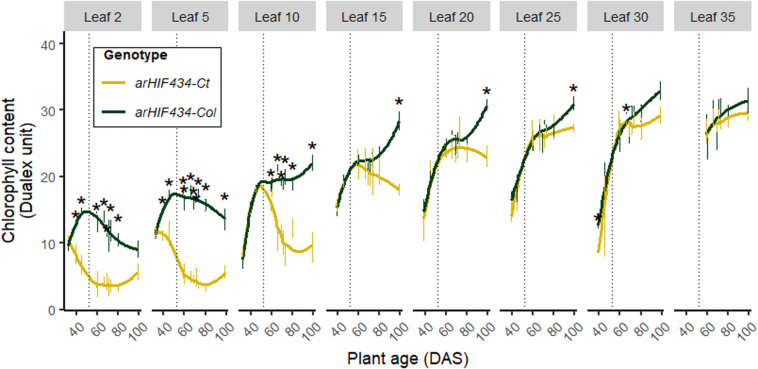
ACD6 activity affects kinetics of chlorophyll content. Yellow and green colors indicate values for *arHIH434-Ct* and *arHIF434-Col*, respectively. Kinetics of chlorophyll content among several leaf ranks in the two *arHIF434* genotypes. Points indicate the average of chlorophyll content (±SE). Stars indicate significant difference between the two genotypes (Student’s test, 4 ≤ *n* ≤ 8, *p* ≤ 0.05). Vertical dotted lines show the flower bud emergence at 52 days after sowing (DAS).

The chlorophyll content of the different rosette leaves were monitored for both *arHIF434* lines during the entire life span of plants (Figure 3). In both genotypes, the chlorophyll content increased with leaf rank. The start of its decrease marked the onset of leaf senescence. In *arHIF434-Col* oldest leaves (rank <10), the chlorophyll content decrease was concurrent with the flower bud emergence, corresponding to a direct effect of the monocarpic leaf senescence. However, in the *arHIF434-Ct* oldest leaves (rank <10), the chlorophyll content decreased before the flower bud emergence, even though this decrease was more pronounced after the floral transition. The significant difference in chlorophyll content between the two genotypes before and after the flower bud emergence indicated that *SEN4* QTL regulated both sequential and monocarpic senescence.

**FIGURE 4:**
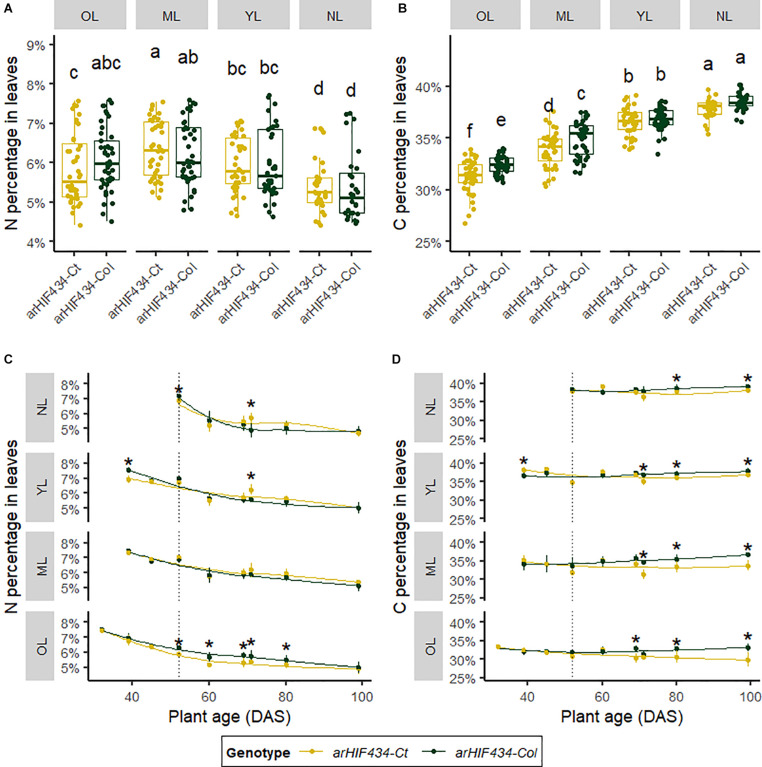
ACD6 activity acts on mobilization of nitrogen and carbon depending on rosette leaf rank. Yellow and green colors indicate values for *arHIH434-Ct* and *arHIF434-Col*, respectively. **(A)** Nitrogen and **(B)** carbon percentages in the groups of leaves in the two *arHIF434* genotypes. Leaves were gathered by groups of 10 leaves: old leaves (OL), ranks 1–10; mature leaves (ML), ranks 11–20; young leaves (YL), ranks 21–30; and new leaves (NL), ranks 31 to >40. Different letters indicate significant difference (Tukey’s test, 30 ≤ *n* ≤ 48, *p* ≤ 0.05). Kinetics of nitrogen **(C)** and carbon **(D)** percentages in the two *arHIF434* genotypes for the four groups of leaves during plant development. Stars indicate significant difference between the two genotypes (Student’s test, 4 ≤ *n* ≤ 8, *p* < 0.05). Vertical dotted lines in panels **(C,D)** show the flower bud emergence at 52 days after sowing (DAS).

The N and C percentages were measured in four group of leaves: (1) old leaves (OL), ranks 1–10; (2) mature leaves (ML), ranks 11–20; (3) young leaves (YL), ranks 21–30; and (4) new leaves (NL), ranks 31 to >40. Independently of the genotype, the average N percentage was higher in the OL, ML, and YL than in NL (Figure 4A). In contrast, the average C percentage increased from OL to NL (Figure 4B). There was no significant variation in the average N percentage between the two *arHIF434* lines irrespective of the leaf group (Figure 4A). However, the average C percentage was lower in *arHIF434-Ct* than in *arHIF434-Col* in the OL and ML groups, which corresponded to the most senescent leaves (Figure 4B). Such differences in element composition between the two genotypes could be due to the effect of *ACD6* on leaf growth or nutrient mobilization.

The N and C percentages showed different kinetics during plant development (Figures 4C,D). The N percentage decreased slowly with plant age, starting on average from 7.5% to reach a plateau at 4.5% (Figure 4C). We noticed a genetic variation in the N percentage kinetic only for OL, in which the decrease in N percentage was faster in *arHIF434-Ct* than in *arHIF434-Col*. The C percentages varied among groups of leaves from 32.3% on average for OL to 38.1% on average for NL. In all leaf groups, the C percentage slightly decreased with plant age in *arHIF434-Ct*, while it increased in *arHIF434-Col* (Figure 4D). The genetic differences in C and N percentage kinetics revealed that *SEN.4* QTL affected the nutrient remobilization from senescing leaves to new organs.

### ACD6 Activity Modulates Rosette Biomass

In a previous study, Chardon et al. (2014) showed that leaf senescence was negatively correlated with rosette, stem, and seed biomass in the *Ct-1* × *Col-0* population and that the *metaQTL4.2* (overlapping the *SEN.4* QTL) had a positive effect on leaf senescence and a negative effect on rosette, stem, and seed biomass. Furthermore, Todesco et al. (2010) have previously shown that a hyperactive allele of *ACD6* reduces leaf biomass.

**FIGURE 5:**
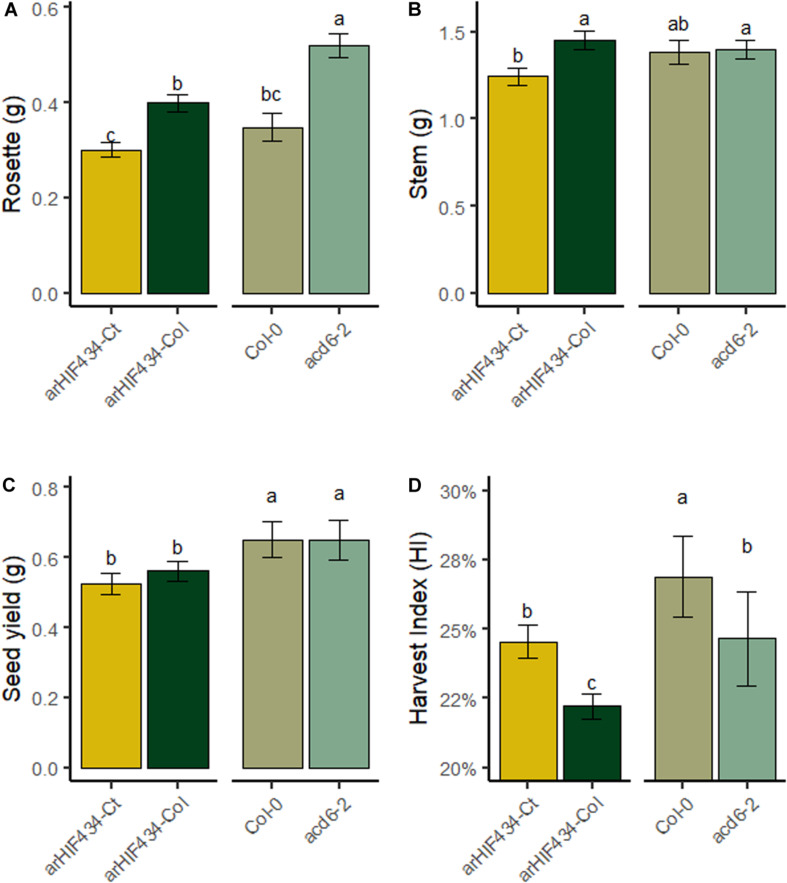
ACD6 activity impacts harvest index by modulating rosette biomass. The dry weight (DW) of **(A)** rosette, **(B)** stem, and **(C)** seeds was measured at harvest and **(D)** the harvest index calculated as the seed DW divided by total plant DW for the four genotypes (*arHIF434-Ct*, *arHIF434-Col*, *Col-0*, and *acd6-2*). Least-square means from two independent experiments ± SE are shown (*n* ≥ 11 for each genotype). Different letters indicate significant difference (Tukey’s test, *p* ≤ 0.05).

In order to investigate the role of *ACD6*, not only during vegetative growth but also during all plant development, the overall DW average variations in three plant compartments, rosette, stem, and seeds (one parameter of plant fitness) were analyzed at maturity in four genotypes (*arHIF434-Ct*, *arHIF434-Col*, *Col-0*, and *acd6-2* mutant) displaying different ACD6 activity (Figure 5). *arHIF434-Ct* displayed a reduced rosette DW compared to *arHIF434-Col*. Conversely, *acd6-2* mutant showed an increased rosette DW compared to *Col-0* (Figure 5A). This result was in accordance with *Ct-1* and *acd6-2* alleles being hyperactive and hypomorphic alleles, respectively, compared to *Col-0* allele. *arHIF434-C*t displayed a reduced stem DW compared to *arHIF434-Col*, but no significant difference in stem DW was observed between *Col-0* and *acd6-2* (Figure 5B). No significant difference in seed DW was observed between the two *arHIF434* genotypes, neither between *Col-0* and *acd6-2* (Figure 5C). As a result, the harvest index (HI), measured as the seed DW divided by total plant DW, was higher in *arHIF434-Ct* compared to *arHIF434-Col* and lower in *acd6-2* compared to *Col-0* (Figure 5D). It is important to mention that no major flowering time difference was observed between *arHIF434-Ct* and *arHIF434-Col*, neither between *Col-0* and *acd6-2* (result not shown).

### ACD6 Enhances Nitrogen Remobilization to Seeds

**FIGURE 6:**
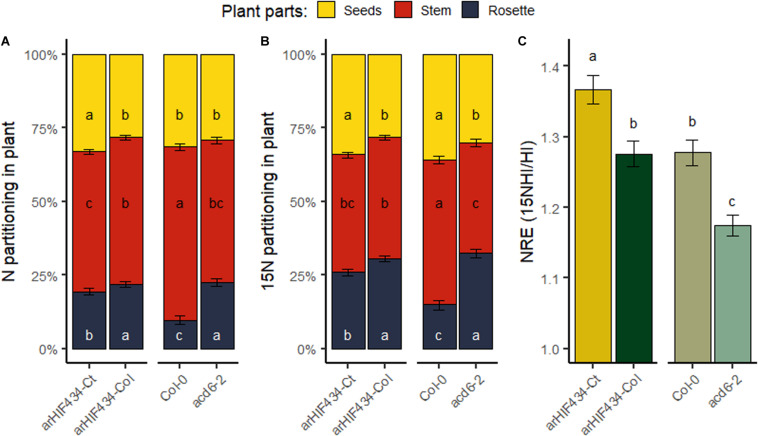
ACD6 modulates N partitioning and N remobilization. **(A)** Proportion of total N in rosette, stem, and seeds in the four genotypes (*arHIF434-Ct*, *arHIF434-Col*, *Col-0*, and *acd6-2*). **(B)** Proportion of total ^15^N in rosette, stem, and seeds in the four genotypes. **(C)** Nitrogen remobilization efficiency for the four genotypes. Least-square means from three independent experiments ± SE are shown. Different letters indicate significant difference between the genotypes (Tukey’s test, *n* ≥ 18, *p* ≤ 0.05).

N partitioning between plant organs at the end of the plant’s life was investigated (Figure 6A). *arHIF434-Ct* and *Col-0* plants allocated more nitrogen to their seeds compared to *arHIF434-Col* and *acd6-2* plants, respectively, even though the difference is not significant between Col-0 and *acd6-2*. However, we did not find a clear impact of *ACD6* on seed quality (Supplementary Figure 4). In addition, the proportion of N in rosette was lower in *arHIF434-Ct* and *Col-0* compared to *arHIF434-Col* and *acd6-2* plants, respectively (Figure 6). Similarly, *arHIF434-Ct* showed lower C percentage in rosette than *arHIF434-Col* (Supplementary Figure 4). These modifications of N partitioning in plants, and the strong variation in nutrient mobilization in rosette (Figures 4A,C), suggested together that ACD6 activity modifies N fluxes in plants.

To better understand the observed differences in N allocation, ^15^N labeling experiments were performed. The ^15^N labeling was applied before bolting, allowing to measure N remobilization from rosette leaves to inflorescence stems and seeds, with the proportion of ^15^N found in the different plant parts (Havé et al., 2016). In both genetic backgrounds (*arHIF434* and *Col-0*), the most active allele of *ACD6* enhanced the proportion of ^15^N in seeds (^15^NHI) and reduced the proportion of ^15^N in rosette compared to the less active allele (Figure 6B). We concluded that ACD6 enhanced plant capacity to remobilize N from rosette to seeds. The N remobilization efficiency (NRE), measured as ^15^NHI on HI ratio, was higher in *arHIF434-Ct* and *Col-0* compared to *arHIF434-Col* and *acd6-2* plants, respectively (Figure 6C). These results demonstrated that ACD6 activity modulates N remobilization efficiency in plants; the more the ACD6 activity, the higher the level of remobilization.

### ACD6 Does Not Affect Nitrate Uptake Efficiency but Enhances N Translocation to Silique

**FIGURE 7:**
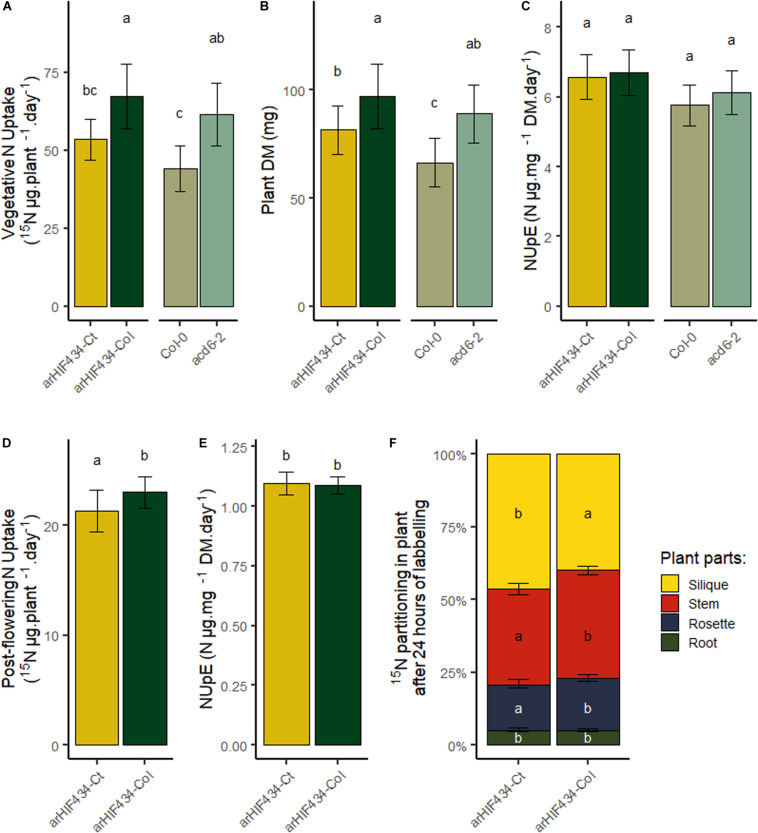
ACD6 does not affect N nitrate uptake efficiency but enhances nitrogen translocation from root to silique. **(A)** Vegetative N uptake in the two *arHIF434* genotypes, *Col-0* and *acd6-2*, as quantity of ^15^N absorbed during 24 h. **(B)** Plant dry matter in the four genotypes. **(C)** Vegetative N uptake efficiency of the four genotypes. **(D)** Postflowering N uptake in the two *arHIF434*, as quantity of ^15^N absorbed during 24 h. **(E)** Postflowering N uptake efficiency in the two *arHIF434*. **(F)** Nitrogen translocation from the roots to the other plant parts. Least-square means from two independent experiments ± SE are shown (*n* = 15 for each genotype). Different letters indicate significant difference between the genotypes (Tukey’s test, *p* ≤ 0.05).

To complete our analysis of ACD6 effect on N fluxes in plants, nitrate uptake capacity of plants during the vegetative and reproductive phases was analyzed. After 24 h of ^15^N labeling, whole plants were harvested, and ^15^N content in roots and rosette was measured. The *arHIF434-Ct* and *Col-0* had absorbed less ^15^N than *arHIF434-Col* and *acd6-2*, respectively (Figure 7A). Since the two last genotypes displayed higher plant weight than the two first ones (Figure 7B), the resulting nitrate uptake efficiencies (NUpE), computed as the ratio between ^15^N quantity absorbed and the biomass of plant, were similar in all the genotypes (Figure 7C). Moreover, no variation in ^15^N transfer between old and new leaves was measured between the genotypes at this stage (Supplementary Figure 5). Then, we estimated the postflowering N uptake in the two *arHIF434* lines. Like for N uptake during the vegetative phase, *arHIF434-Ct* absorbed less ^15^N than *arHIF434-Col* in 24 h (Figure 7D), but the NUpE of both genotypes were similar (Figure 7E) since *arHIF434-Ct* is smaller than *arHIF434-Col*. Interestingly, the proportion of ^15^N stored in silique was more important in *arHIF434-Ct* than in *arHIF434-Col* (Figure 7F). Simultaneously, *arHIF434-Col* transferred more nitrate from the roots to inflorescence stems and rosette than *arHIF434-Ct*. We concluded that an enhanced ACD6 activity promoted the translocation of nitrogen from root to silique.

## Discussion

### ACD6 Regulates Natural Senescence Process Before and After the Flowering Time

Leaf senescence is a crucial process for nutrient mobilization and recycling from old organs to support the growth of new organs. Previously, we detected a locus, *SEN.4*, involved in a large variation in leaf senescence between *Col-0* and *Ct-1* accessions (Chardon et al., 2014). Here, we fine mapped the locus to a small genomic region including two genes, *ACL1* and *ACD6* (Figure 1C). The first one is nearly not expressed in plant (eFP Browser, Winter et al., 2007). It was indeed undetectable in wild-type *Col-0* leaves. The latter is specifically expressed in leaves (eFP Browser, Winter et al., 2007). Furthermore, *ACD6* is expressed during the entire leaf lifespan but in an age-dependent manner (Andriankaja et al., 2012; Woo et al., 2016). The absence of *ACD6* gene expression in the corresponding *acd6-2* knockout mutant (Figure 2B) delays rosette leaf senescence. On the contrary, the senescence of the *acl1-1* mutant is not affected compared to *Col-0* (Figure 2C), in accordance with the phenotype of a KO mutant with artificial microRNA (Todesco et al., 2014). Together, the fine mapping and the phenotype of the mutants demonstrated that *ACD6* regulates the natural leaf senescence process and that its polymorphism is involved in the leaf senescence variation observed between *Col-0* and *Ct-1* accessions.

*ACD6* gene encodes a protein with ankyrin and transmembrane domains (Lu et al., 2003). The spontaneous leaf necrosis phenotype of the gain of function mutant gave the name of the gene: *ACCELERATED CELL DEATH 6* (Lu et al., 2003). Little is known about the molecular function of ACD6 protein (Lu et al., 2005). Nevertheless, it has been demonstrated that the ACD6 protein plays a major role in plant response to biotic and abiotic stresses through the SA signaling pathway (Rate et al., 1999; Todesco et al., 2014; Pluhařová et al., 2019). During pathogen infection, the rise in SA levels in leaves triggers the cell death program and leaf necrosis. Todesco et al. (2010) revealed that the natural variation in *ACD6* expression affects both leaf initiation rate and late-onset leaf necrosis by using the diversity of *Arabidopsis* accessions. The hyperactive *ACD6* allele of *Est-1* accession, a closely related allele of *Ct-1*, reduced the leaf initiation and induced extensive and spontaneous necrosis development on fully expanded leaves. Interestingly, *ACD6* is also involved in the hybrid incompatibility generated by crossing two genotypes from the same species (Todesco et al., 2014; Ṡwiadek et al., 2017). Specific combinations of *ACD6* alleles caused natural hybrid necrosis resulting in spontaneous activation of plant defenses associated with leaf cell death, reduced growth, accumulation of SA, and low fertility in hybrids. The geographic dispersion of natural *ACD6* alleles, sometimes incompatible, that enhances either plant defense or leaf growth support the hypothesis that the *ACD6* locus is involved in an adaptive trade-off in *Arabidopsis* (Todesco et al., 2014; Ṡwiadek et al., 2017). In this study, the analysis of four genotypes displaying three *ACD6* alleles—the hyperactive *Ct-1*, the *Col-0* or the hypomorphic *acd6-2* allele—was congruent with this role of *ACD6*. Indeed, the *Ct-1* hyperactive allele of *ACD6* promoted the natural leaf senescence but reduced the rosette and stem inflorescence DW compared to the *Col-0* allele (Figures 1, 5). On the contrary, the *acd6-2* hypomorphic allele of *ACD6* slowed natural leaf senescence down but increased rosette DW (Figures 2, 5).

During the *Arabidopsis* lifespan, two phases of senescence can be distinguished (Schippers et al., 2015). During the first one, occurring before the flower bud emergence, the sequential leaf senescence appears sequentially from the older basal rosette leaves to the younger apical ones. Then, the stem inflorescence development and seed production lead to the need of rosette compound recycling. Consequently, the development of seeds enhanced the senescence of all rosette leaves in *Arabidopsis*. Previous observations of *ACD6* allelic variation report leaf necrosis during vegetative stage. Following chlorophyll content kinetic before and after the flower bud emergence, we provided evidence that ACD6 regulates both the sequential and monocarpic senescence (Figure 3).

### ACD6 Modulates Finely the Nitrogen Remobilization Efficiency

In plants, senescence is a dynamic process with several phases in which the nutrients, especially N-rich compounds, are remobilized from the senescing organs to the new ones (Malagoli et al., 2005; Diaz et al., 2008; Lemaître et al., 2008). It is a finely regulated genetic process involving a coordinated action at the cellular, tissue, organ, and organism levels (Lim et al., 2007). A complex network of regulatory pathways fine tunes the timing of the plant senescence in response to both external and internal clues, such as plant pathogen, nutrient starvation, and phytohormones, including abscisic acid, jasmonic acid, ethylene, and SA. For instance, SA levels in leaves participates to the natural senescence by regulating the expression of genes that are also modified by abiotic stresses (Morris et al., 2000; Lim et al., 2007). Following the N percentage in leaves and the N remobilization from the leaves to the seeds, we provided evidence that ACD6 acts on N mobilization during leaf senescence (Figures 4A,C). We showed that ACD6 also increased by 10% the N remobilization to seeds during the reproductive period (Figure 6). Other cellular processes have been previously shown to act simultaneously on leaf senescence and N remobilization to seeds. For instance, defect in the macroautophagy process, an intravesicular process for vacuolar bulk degradation of cytoplasmic components, enhanced leaf senescence but limited N remobilization efficiency. Knockout mutants of autophagy genes conserved only around 40% of their N remobilization efficiency in *Arabidopsis* when the plants were grown in low N condition (Guiboileau et al., 2012). Similarly, a maize mutant affected in the autophagy process displayed only 60% of the N remobilization efficiency of the wild type (Li et al., 2015). Moreover, cytosolic glutamine synthetases, key enzymes of ammonium assimilation during the N recycling process, are induced during leaf senescence (Diaz et al., 2008; Lothier et al., 2011) and act on N remobilization efficiency (Moison et al., 2018). The *gln1;1-gln1;2-gln1;3* triple mutant displayed a 12% reduction in N remobilization to seeds compared to wild type (Moison et al., 2018). In addition, environmental stresses may also have a large effect on N remobilization to seeds, which is increased by 38% under N limited condition or reduced by 45% under heat stress (Marmagne et al., 2020). In this context, we concluded that ACD6 has a major effect on natural senescence but plays a limited role on N remobilization efficiency compared to other cellular processes and environmental stresses.

### Advantage and Limitation of a Fast Leaf Senescence Onto N Remobilization Capacity

*ACCELERATED CELL DEATH 6* had a positive effect on leaf senescence as well as on N remobilization efficiency of plants although to a lesser extent (Figures 4, 6). Yet, the effect of the locus onto the seed composition was very limited (Supplementary Figure 4). Our results highlighted two phenomena occurring during seed filling: (i) the negative impact of excessive leaf senescence on N mobilization process and (ii) the balance between N remobilization and uptake during the reproductive phase.

*ACD6* induced a burst of SA levels in cells leading to a rapid cell death and resulting in leaf necrosis as reported by several authors (Lu et al., 2003; Todesco et al., 2014). In our conditions, the plants with a hyperactive *Ct-1 ACD6* allele showed a faster senescence of rosette than the *Col-0* plants (Figures 1A,B, 3A). A fast senescence process might be an asset for the plant to isolate a pathogen infection from the healthy parts of the leaves and to activate concomitantly the plant defense system. An early onset of leaf senescence might also help in mobilizing leaf nutrients. However, even though all the leaves from the *Ct-1* plants senesced more rapidly than the leaves from the *Col-0* ones, we observed that *ACD6* effect on N mobilization (i.e., decrease of N percentage in leaves) varied among leaf ranks (Figure 4C). Indeed, the oldest leaves showed the strongest response to *ACD6* variation for N mobilization compared to the youngest and newest leaves. We noticed that variation in N mobilization is associated to the difference in sequential senescence in the old leaves (Figure 4C). Nevertheless, the strong mobilization of N induced by seed filling after flower bud emergence did not correlate with the difference in monocarpic senescence between the two genotypes (Figure 3). After flower bud emergence, N percentage decreased in medium, young, and new leaves, in contrast to the chlorophyll content (Figure 3), highlighting that the mobilization of N compounds occurs before the monocarpic senescence. Similarly to rice, mobilization of metabolites from the flag and second leaves occurs before chlorophyll decrease during grain filling (Ray and Choudhuri, 1981; Lee et al., 2017). We assumed that if leaf senescence is early but too intense, the N mobilization process could be interrupted due to the rapid death of the leaves.

The N stored in seed is derived from direct N uptake from the soil and N recycling from other organs during the reproductive phase (Masclaux-Daubresse et al., 2010). Growing and storage organs are two elements that drive N transportation within plants (Yoneyama et al., 2003). Likewise, the source–sink relationship created by seed production is the main driver of N remobilization efficiency in *Arabidopsis* (Masclaux-Daubresse and Chardon, 2011). In the present study, the use of ^15^N-labeled nitrate allowed us to estimate postflowering N uptake and remobilization of plants. We showed that *ACD6* acts on the N remobilization efficiency (Figure 6) but does not affect the N uptake efficiency, during neither vegetative nor reproductive stages (Figures 7C,E). However, *ACD6* impaired N fluxes and N translocation during the reproductive phase (Figure 7F). In particular, *ACD6* activity enhanced the translocation of nitrogen from root to silique. We assumed then that the death of several leaves, due to the action of *ACD6*, limits transitory storage of N in rosette. Following this hypothesis, the N requirements for seed filling are fulfilled by N uptake in hyperactive *Ct-1 ACD6* allele, reducing the strength of the sink for N remobilization. Consequently, the positive effect of *ACD6* onto N remobilization due to early leaf senescence is partially balanced by the negative and indirect effect of *ACD6* on N uptake. Similar compensatory phenomenon between N uptake and N mobilization have been observed in maize in which an accelerated leaf senescence results in a decrease in source–sink ratio and a reduction in the proportion of N in the grain that was taken up during grain filling (Rajcan and Tollenaar, 1999).

We observed that leaf senescence was negatively correlated to N percentage in seeds in different recombinant inbreed line populations, in particular the *Ct-1* × *Col-0* one (Chardon et al., 2014). Several hypotheses could explain the difference between the QTL effect on N percentage in seeds in the *Ct-1* × *Col-0* population and the lack of effect detected in the present study (Supplementary Figure 4). First, the genetic regulation of seed filling is complex, and different genes could be located in the same genomic region and act independently on leaf senescence and seed filling. Second, because seed filling is sensitive to the environment (Marmagne et al., 2020), the small environmental variations inherent to the different experiments may change the regulation of the seed filling process. Third, because the senescence process is also influenced by a range of environmental factors, such as low nutrient supply, photoperiod, temperature, and drought (Lim et al., 2007; Schippers et al., 2015; Santos Matos, 2020), small variations in the environment may affect the leaf senescence intensity promoted by the hyperactive *ACD6* allele. Several genetic analyses pointed out that both the onset and the duration of leaf senescence act on the grain filling in crop plants (Hafsi et al., 2000; Gregersen et al., 2013; Xie et al., 2016; Kitonyo et al., 2018). For instance, Xie et al. (2016) showed that a delayed but fast leaf senescence promoted grain-filling rates in bread wheat. These results were in accordance with our hypothesis that the duration and intensity of leaf senescence act on the N mobilization process. In addition to the trade-off opposing plant growth and plant defense associated to *ACD6* reported by Todesco et al. (2014), we bring here a new link showing the extra level of regulation of *ACD6* on leaf senescence and nutrient use efficiency.

## Data Availability Statement

The raw data supporting the conclusions of this article will be made available by the authors, without undue reservation.

## Author Contributions

SJ, IF, AmM, and AL performed the QTL fine mapping. SJ performed the phenotyping and molecular analysis of the T-DNA mutants, as well as the *ACD6* sequences analysis. FC and SJ did the chlorophyll measurement. AnM performed C and N percentage analyses and ^15^N isotopic measurements. AnM and SJ performed the q-RT-PCR. FC carried out the statistical analysis. FC and SJ designed the research, analyzed the data, and wrote the manuscript. All authors read and approved the final manuscript.

## Conflict of Interest

Since August 2019, the co-author AmM has been employed by Frontiers Media SA. AmM declared their affiliation with Frontiers and the handling editor states that the process nevertheless met the standards of a fair and objective review. The remaining authors declare that the research was conducted in the absence of any commercial or financial relationships that could be construed as a potential conflict of interest.

## Footnotes

1 https://www.r-project.org/

## Supplementary Material

Supplementary Figure 1 *arHIF434-Ct* displays an earlier leaf senescence than *arHIF434-Col*. **(A)** *arHIF434* is represented with horizontal bars (black for *Col-0* allele, white for *Ct-1* allele, grey for heterozygous). Dashed vertical bars represent markers delimiting the candidate interval on chromosome 4. Numbers correspond to marker position (Mb). Position of *ACL1* and *ACD6* genes are shown above the arHIF. **(B)** Rosette leaves of 5-week-old plants. Upper rows: *arHIF434-Ct*, lower rows: *arHIF434-Col*. Scale bar corresponds to 1 cm. **(C)** Percentage of senescent leaves in *arHIF434-Ct* (yellow triangle line) and *arHIF434-Col* (dark green circle line) during the reproductive phase. Flowering transition occurred in average at 23.7 DAS for *arHIF434-Col* and 23.9 DAS for *arHIF434-Ct*.

Supplementary Figure 2 Protein sequence alignment of ACD6 from *Col-0* and *Ct-1* accessions. Numbers indicate amino acid position from the first Methionine. Grey box correspond to ankyrin repeats and black lines to transmembrane domains as predicted using SMART website (http://smart.embl-heidelberg.de/). ^∗^Amino acids 566 and 634.

Supplementary Figure 3 Expression of *ACD6* in *arHIF434-Ct* and *arHIF434-Col*. Plants were grown under long days (8 h light/16 h dark) for 35 d after sowing and then harvested. Transcript levels of *ACD6* **(A)***; SAG12* **(B)**, and *RBCS1A* **(C)** marker genes of leaf senescence, and *PR1* **(D)** involved in SA signaling process, were monitored using RT–qPCR and specific primers (Supplementary Table 1). Expression of *ACD6* was normalized using *PP2AA3* and *APC2*. Expression of *PR1*, *SAG12* and *RBCS1A* were normalized using *PP2AA3.*

Supplementary Figure 4 Effect of *ACD6* on N and C percentages in the different parts of the plants. N and C percentages in rosette **(A,B)**, stem **(C,D)**, and seeds **(E,F)**. N and C percentages for the four genotypes (*arHIF434-Ct*, *arHIF434-Col*, *Col-0* and *acd6-2*) are shown. Least-square means from 3 independent experiments ± s.e. are shown (*n* ≥ 18 for each genotype). Different letters indicate significant difference (Tukey’s test, *p*-value ≤ 0.05).

Supplementary Figure 5 ACD6 does not affect N translocation (T1) and remobilization (T2) from old leaves to young leaves during the vegetative phase. The four genotypes (*arHIF434-Ct*, *arHIF434-Col*, *Col-0* and *acd6-2*) were grown on sand in short day conditions (8 hours). After 48h of labeling with ^15^NO3, lower (ranks 1 to 10) and upper (ranks > 10) leaves were harvested and grouped. Proportion of total ^15^N was measured in the two groups of leaves, just after the labeling period (T1) to estimate the N translocation, and 7 days after (T2) to estimate the N remobilization from old leaves to young leaves during vegetative phase.
