# Supplementary material for: ACCELERATED CELL DEATH 6 Acts on Natural Leaf Senescence and Nitrogen Fluxes in Arabidopsis
Source: Front Plant Sci. 2021 Jan 7;11:611170. doi: 10.3389/fpls.2020.611170 (PMC7817547; doi:10.3389/fpls.2020.611170)
Supplement: Supplementary Figure 1 — arHIF434-Ct displays an earlier leaf senescence than arHIF434-Col. (A) arHIF434 is represented with horizontal bars (black for Col-0 allele, white for Ct-1 allele, grey for heterozygous). Dashed vertical bars represent markers delimiting the candidate interval on chromosome 4. Numbers correspond to marker position (Mb). Position of ACL1 and ACD6 genes are shown above the arHIF. (B) Rosette leaves of 5-week-old plants. Upper rows: arHIF434-Ct, lower rows: arHIF434-Col. Scale bar corresponds to 1 cm. (C) Percentage of senescent leaves in arHIF434-Ct (yellow triangle line) and arHIF434-Col (dark green circle line) during the reproductive phase. Flowering transition occurred in average at 23.7 DAS for arHIF434-Col and 23.9 DAS for arHIF434-Ct. [file Presentation_1.pdf]

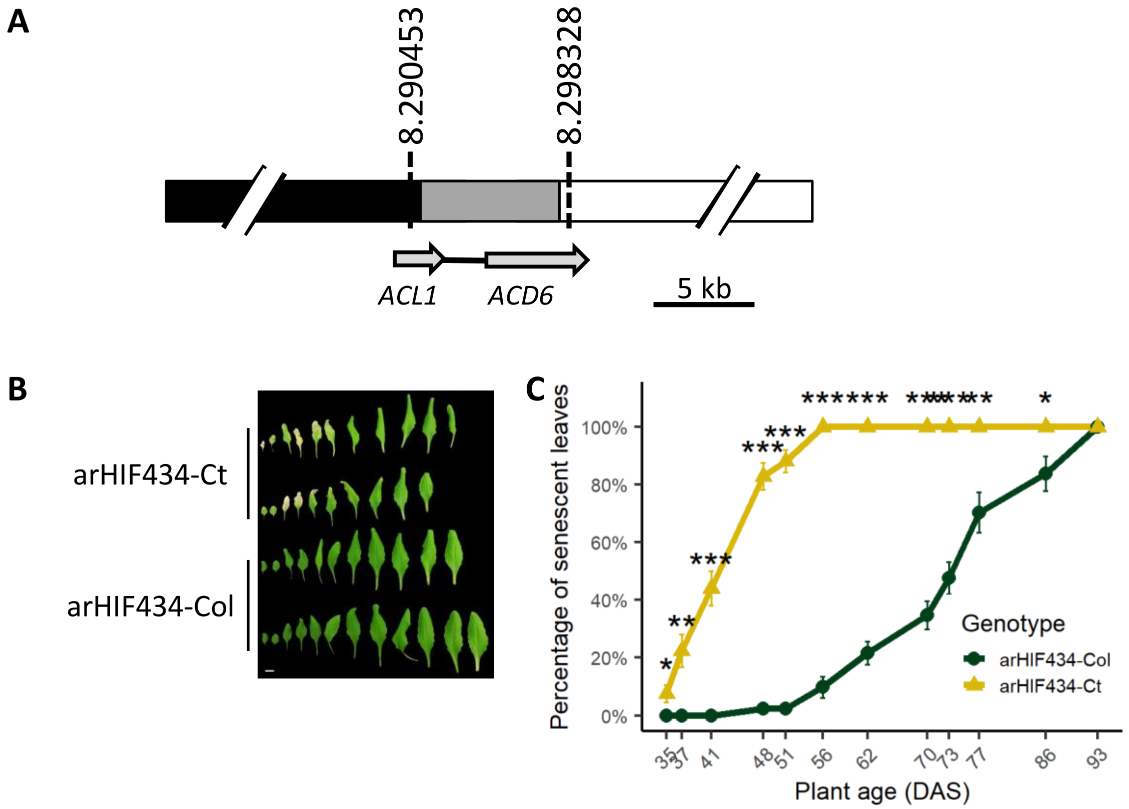

Supplementary FIGURE 1. *arHIF434-Ct* displays an earlier leaf senescence than *arHIF434-Col*. **(A)** *arHIF434* is represented with horizontal bars (black for *Col-0* allele, white for *Ct-1* allele, grey for heterozygous). Dashed vertical bars represent markers delimiting the candidate interval on chromosome 4. Numbers correspond to marker position (Mb). Position of *ACL1* and *ACD6* genes are shown above the arHIF. **(B)** Rosette leaves of 5-week-old plants. Upper rows: *arHIF434-Ct*, lower rows: *arHIF434-Col*. Scale bar corresponds to 1 cm. **(C)** Percentage of senescent leaves in *arHIF434-Ct* (yellow triangle line) and *arHIF434-Col* (dark green circle line) during the reproductive phase. Flowering transition occurred in average at 23.7 DAS for *arHIF434-Col* and 23.9 DAS for *arHIF434-Ct*.

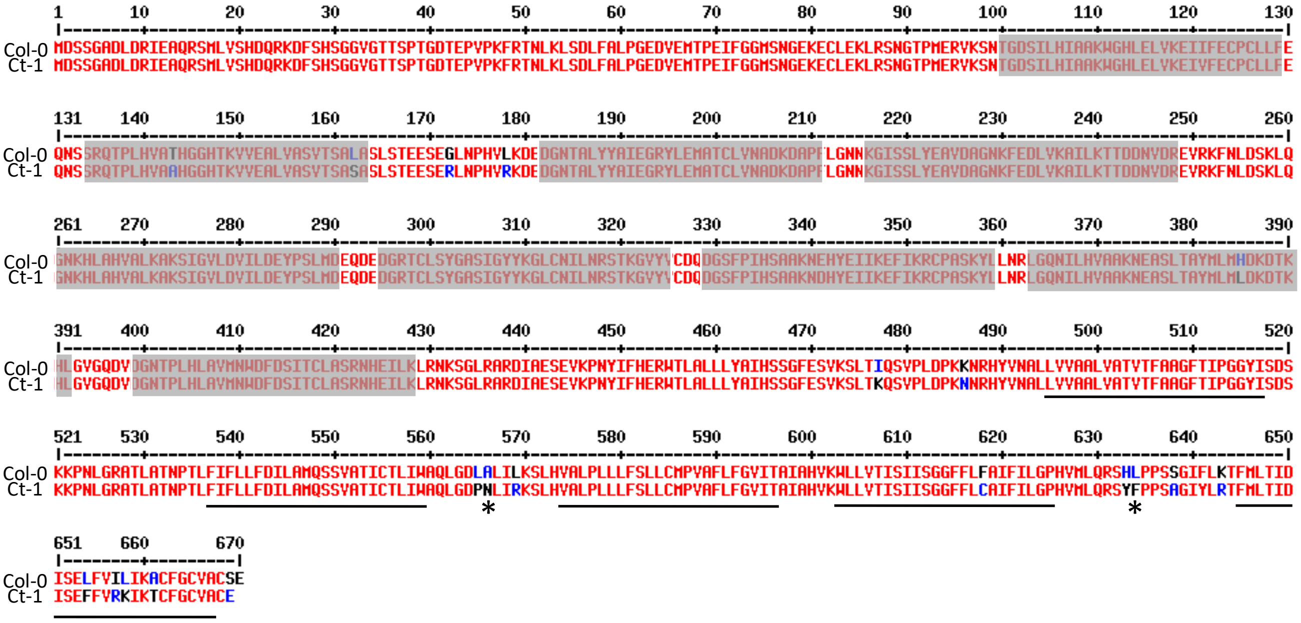

Supplementary FIGURE 2. Protein sequence alignment of ACD6 from *Col-0* and *Ct-1* accessions. Numbers indicate amino acid position from the first Methionine. Grey box correspond to ankyrin repeats and black lines to transmembrane domains as predicted using SMART website (http://smart.embl-heidelberg.de/).
* Amino acids 566 and 634.

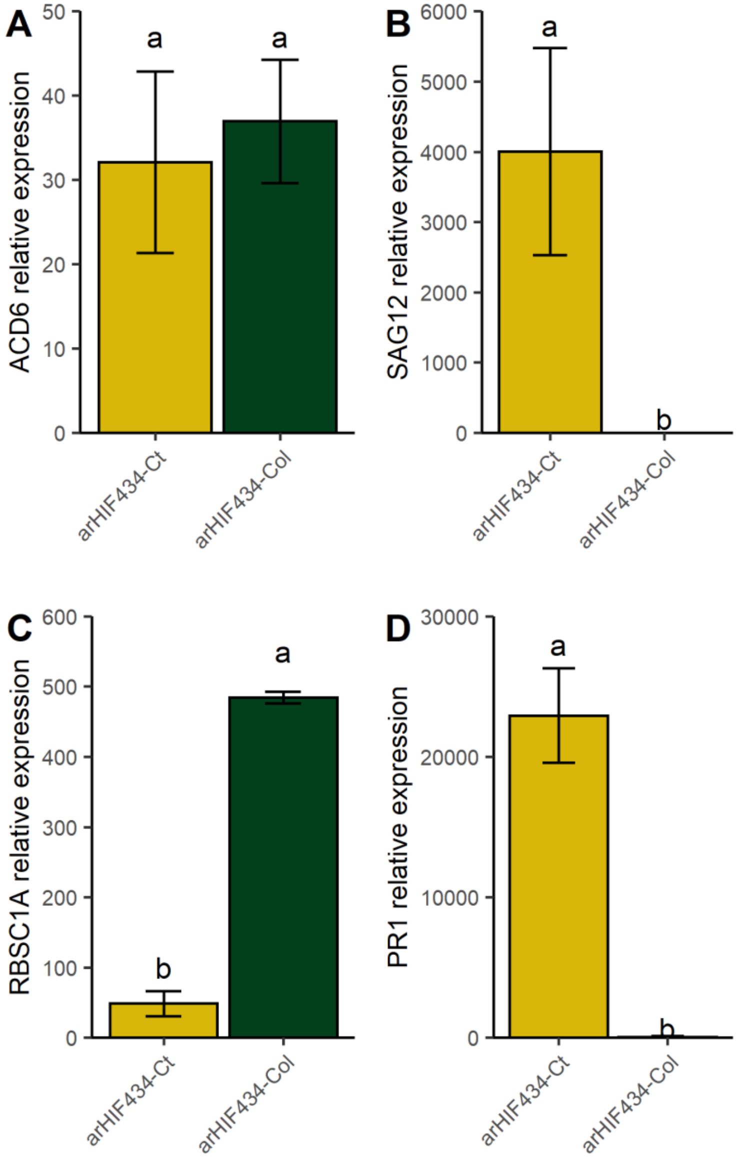

Supplementary FIGURE 3. Expression of *ACD6* in *arHIF434-Ct* and *arHIF434-Col*. Plants were grown under long days (8 h light/16 h dark) for 35 d after sowing and then harvested. Transcript levels of *ACD6* **(A)***; SAG12* **(B)** and *RBCS1A* **(C)** marker genes of leaf senescence, and *PR1* **(D)** involved in SA signaling process, were monitored using RT–qPCR and specific primers (Supplementary Table S1). Expression of *ACD6* was normalized using *PP2AA3* and *APC2*. Expression of *PR1*, *SAG12* and *RBCS1A* were normalized using *PP2AA3.*

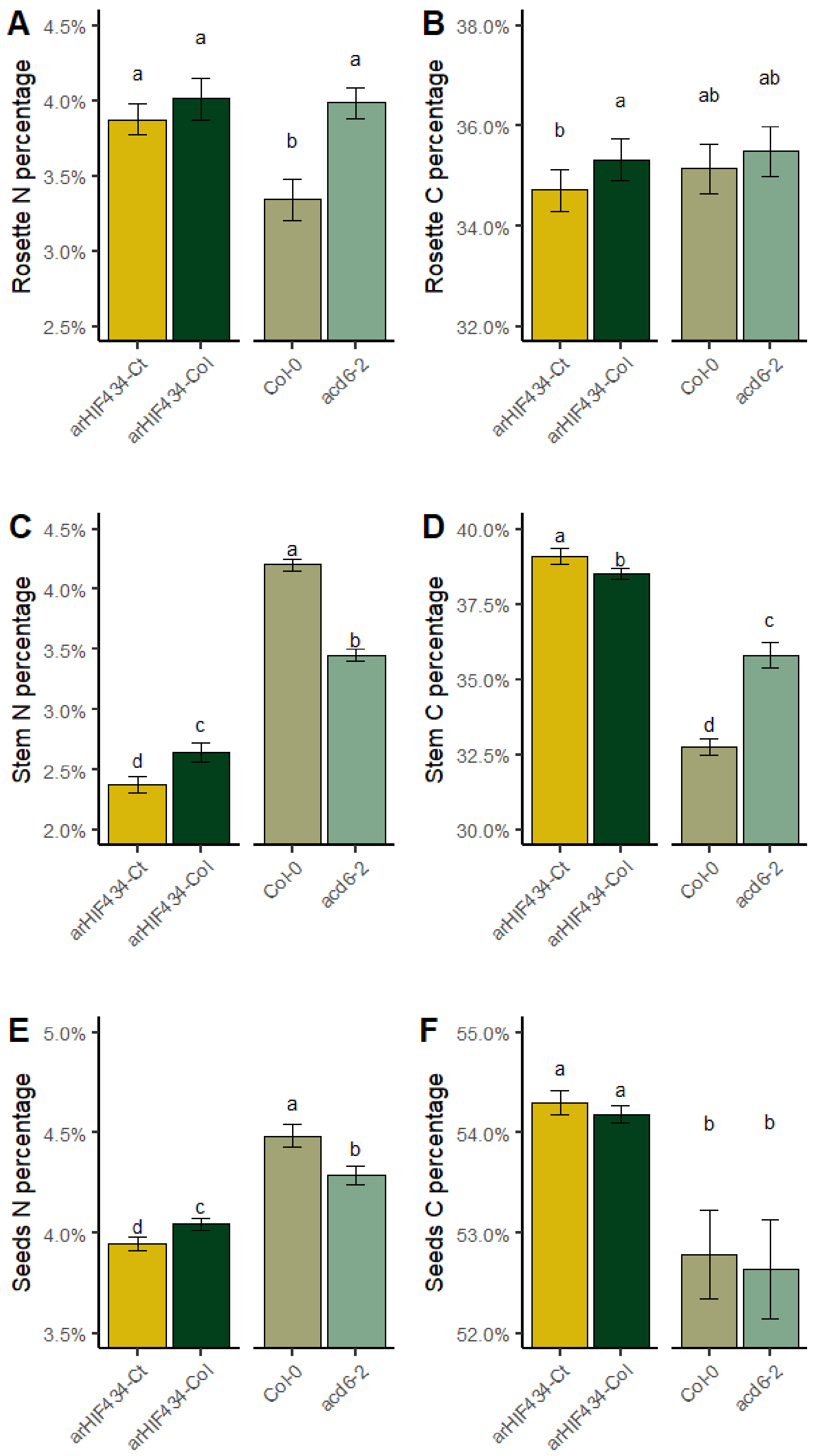

Supplementary FIGURE 4. Effect of *ACD6* on N and C percentages in the different parts of the plants. N and C percentages in rosette **(A,B)**, stem **(C,D)**, and seeds **(E,F)**. N and C percentages for the four genotypes (*arHIF434-Ct*, *arHIF434-Col*, *Col-0* and *acd6-2*) are shown. Least-square means from 3 independent experiments ± s.e. are shown (n ≥ 18 for each genotype). Different letters indicate significant difference (Tukey's test, p-value ≤ 0.05).

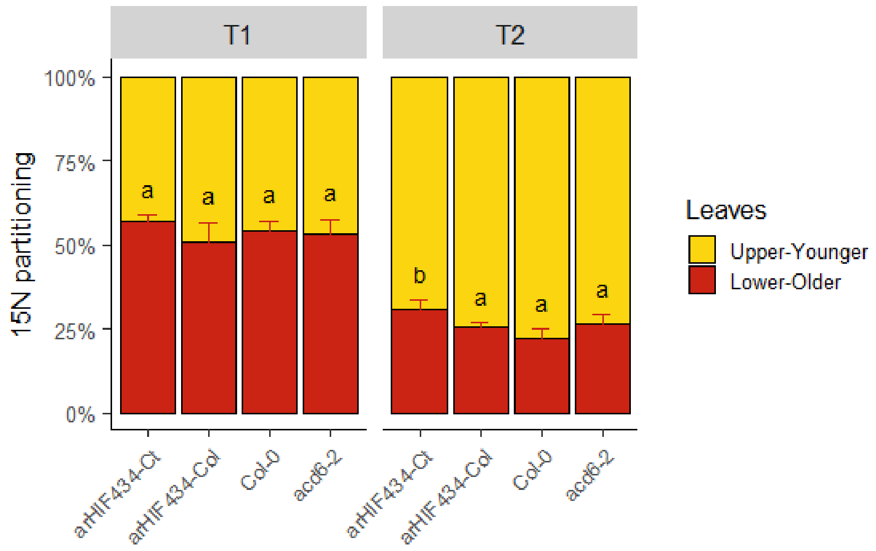

Supplementary FIGURE 5. ACD6 does not affect N translocation (T1) and remobilization (T2) from old leaves to young leaves during the vegetative phase. The four genotypes (*arHIF434-Ct*, *arHIF434-Col*, *Col-0* and *acd6-2*) were grown on sand in short day conditions (8 hours). After 48h of labelling with $^{15}NO3$, lower (ranks 1 to 10) and upper (ranks >10) leaves were harvested and grouped. Proportion of total ^{15}N is measured in the two groups of leaves, just after the labelling period (T1) to estimate the N translocation, and 7 days after (T2) to estimate the N remobilization from old leaves to young leaves during vegetative phase.
