# Supplementary material for: ACCELERATED CELL DEATH 6 Acts on Natural Leaf Senescence and Nitrogen Fluxes in Arabidopsis
Source: Front Plant Sci. 2021 Jan 7;11:611170. doi: 10.3389/fpls.2020.611170 (PMC7817547; doi:10.3389/fpls.2020.611170)
Supplement: Supplementary file 2 [file Table_1.pdf]

Supplementary Table S1. Primers used for genotyping, RT-PCR and qRT-PCR analyses

Gene	AGI	Forward Primer	Reverse Primer	Product size (bp)	Purpose
ACD6	AT4G14400	SALK-045869-RP2: CGCTGGAAATAAATTTGAAGAC	SALK-045869-LP2: CGTAATTTCAGTATCTCGTGGT	740	*acd6-2* genotyping
ACL1	AT4G14390	GABI_108H02-RP: TTTACGCTATCCGTGGTGAAG	GABI_108H02-LP2: TATACATACAAATATTTTTACCGC	759	*acl1-1* genotyping
ACD6	AT4G14400	ACD6-F1: TGCACATGTTGCTTTGAAGG	SeqACD6-R5: TTTTGATGATTTATTCGGAACAC	1229	RT-PCR
ACL1	AT4G14390	At4g14390-For1: CTCAATCTCGTCAACCTTTAG	At4g14390-Rev1: TTAATAACAACCACAAAAAAGAA	2068	RT-PCR
ACT2	AT3G18780	ActQ1F: GCCATCCAAGCTGTTCTCTC	ActQ2R: CCCTCGTAGATTGGCACAGT	101	RT-PCR
ACD6	AT4G14400	ACD6-F3: ATCACTGCAATTGCCCATG	ACD6-R3: ACACGCCACACAACCAAAA	222	qRT-PCR
PP2AA3	AT1G13320	PP2A3-For: GCAATCTCTCATTCCGATAGTC	PP2A3-Rev: ATACCGAACATCAACATCTGG	100	reference gene for qRT-PCR
ACP2	AT2G04660	ACP2-For: GAAACATCAATTGCCTCTGTGGAAGA	ACP2-Rev: AAGGATCAGCCACACAAAACATCTTG	148	reference gene for qRT-PCR
SAG12	AT5G45890	SAG12F: CCCGGTTAATGATGAGCAAGC	SAG12R: GCTTTCATGGCAAGACCACA	294	qRT-PCR
PR1	AT2G14610	PR1-F: TCTTCCCTCGAAAGCTCAAGA	PR1-R: GTGCCTGGTTGTGAACCCTTA	60	qRT-PCR
RBCS1A	AT1G67090	RBCS1aF: ATTGCCTACAAGCCACCAAG	RBCS1aR: ATTTGTAGCCGCATTGTCCT	277	qRT-PCR
