# Supplementary material for: ACCELERATED CELL DEATH 6 Acts on Natural Leaf Senescence and Nitrogen Fluxes in Arabidopsis
Source: Front Plant Sci. 2021 Jan 7;11:611170. doi: 10.3389/fpls.2020.611170 (PMC7817547; doi:10.3389/fpls.2020.611170)
Supplement: Supplementary file 3 [file Table_2.pdf]

Supplementary table S2. Markers used for mapping

Marker	Position on chr. 4 (pb)	Marker type	SNP *	Forward primer	Reverse primer	PCR product size in Col-0 (pb)	Restriction enzyme	Col-0 digestion product	Ct-1 digestion product
MSAT4.07180	7.180000	microsatellite	n/a	GTCAGAGCCCAAGTCCAGAG	AAGCTTTTGGGATAACGAATGA	172	n/a	n/a	n/a
MSAT4.07636b	7.636981	microsatellite	n/a	CTCCATTAGAGACATCCTCGG	ACTAACTCGGAGACAAACCGG	325	n/a	n/a	n/a
IND4.07732	7.732971	indel	n/a	GCAGTGAAGTGCAATCCTGA	CTCTGCTCATAGATCCCATACG	294	n/a	n/a	n/a
IND4.07847	7.847209	indel	n/a	TCAATCTTGCGCACTCAAAC	TGAATCTTGGCCGAATAAGG	207	n/a	n/a	n/a
MSAT4.08053	8.050000	microsatellite	n/a	ATTCTCACTGTCGGCTGCTT	GCAATAGCCACCAAGCATTT	243	n/a	n/a	n/a
IND4.08100	8.100321	indel	n/a	TCGCAAACACACAGTGACAA	CAAAACCGGGTCGAATTATG	283	n/a	n/a	n/a
IND4.08139	8.139434	indel	n/a	AGAACTTCCCCATGCTTTTCA	ACGTTTGTCAATTTGCCCAA	136	n/a	n/a	n/a
IND4.08211	8.211624	indel	n/a	CCAATCATAACCCTGCATCCA	TGTTGAAAACTGCTGATCCGA	237	n/a	n/a	n/a
IND4.08252	8.252361	indel	n/a	TCAACAACAACACGAAACAAGT	TCCAGAATCTGTCCAAGCGA	484	n/a	n/a	n/a
MSAT4.08286	8.286213	microsatellite	n/a	CAAAGCGTCGTCAGTATGGA	ACTCGTCGGCTCTCTCGTTA	243	n/a	n/a	n/a
IND4.08288b	8.288836	indel	n/a	ACCGTTGCCTGATTTGAGTC	CATGCAATGCCAACTCTTTG	250	n/a	n/a	n/a
IND4.08288c	8.288840	indel	n/a	ACCGTTGCCTGATTTGAGTC	CATGCAATGCCAACTCTTTG	250	n/a	n/a	n/a
IND4.08288d	8.288991	indel	n/a	TCAGCCAAAGAGTTGGCATT	AGATTCAATGGGATCGCAAA	133	n/a	n/a	n/a
IND4.08289	8.289003	indel	n/a	TCAGCCAAAGAGTTGGCATT	AGATTCAATGGGATCGCAAA	133	n/a	n/a	n/a
SNP4.08290	8.290138	CAPS	G::A	ATAGAATTGAGGCACAAAGGTCA	AATGCAGCTGCTACTGGTTAAAG	863	NcoI	863	275, 588
SNP4.08290b	8.290145	CAPS	T::G	ATAGAATTGAGGCACAAAGGTCA	AATGCAGCTGCTACTGGTTAAAG	863	ApoI	280, 583	863
SNP4.08290c	8.290182	CAPS	A::G	ATAGAATTGAGGCACAAAGGTCA	AATGCAGCTGCTACTGGTTAAAG	863	TasI	396, 276, 148 (39, 4)	711, 148 (4)
SNP4.08290d	8.290453	CAPS	G::A	ATAGAATTGAGGCACAAAGGTCA	CAACCATTTATATCTCGCTCGTC	1032	Bsp143II	593, 439	917, 115
SNP4.08291	8.291555	CAPS	C::T	AGAGAGAGGTGAAACCCCACTAC	GAATGAGCACTGGATCACCTAAC	457	HpaI	457	231, 226
SNP4.08292	8.292439	CAPS	T::G	GCTACAACGGTCATACGTTTCTC	AAATTTTCATGTTTGTTTGGTGG	611	NlaIII, Hin1II	481 (65, 58, 7)	539 (65, 7)
SNP4.08293	8.293857	CAPS	T::G	TCCGATAGTCATAGCCTCAGAAC	CCACCACACACAGAGTTTTCATA	827	HindIII	600, 227	827
SNP4.08296	8.296497	sequencing	A::G	ATGGCTCTGAACGATTGTCT	TTCACCTCTGACTCGGCAAT	2238	n/a	n/a	n/a
SNP4.08297	8.297287	CAPS	A::T	GAAAGCAATTTTGAAAACCACAG	GTGGAGAATGTTCTGACCAAGTC	531	MboI	376, 155	376 (87, 68)
SNP4.08297b	8.297418	sequencing	A::T	ATGGCTCTGAACGATTGTCT	TTCACCTCTGACTCGGCAAT	2238	n/a	n/a	n/a

SNP4.08298	8.298328	CAPS	G::A	ATGGCTTTTAGTCACCATTAGCA	CTCATCTCCTTTGAATGTGGTTC	489	HindIII	315, 174	489
SNP4.08298b	8.298474	CAPS	C::T	ATGGCTTTTAGTCACCATTAGCA	CTCATCTCCTTTGAATGTGGTTC	489	EcoRI	489	319, 170
IND4.08300	8.300260	indel	n/a	TCGTCCTTTCGCACTCTTCT	TCAAGTTCTGCTCCATTGCT	340	n/a	n/a	n/a
IND4.08302	8.301657	indel	n/a	CCGTTTGGCAAGTGTAAAAGC	GAGCACCCGTTTAGTAAGCG	201	n/a	n/a	n/a
IND4.08303	8.302712	indel	n/a	CGAAGGAACAGTTGGTGCAT	TGCCACCAGTCGATGTATGT	223	n/a	n/a	n/a
IND4.08312	8.312050	indel	n/a	TTCGGATTTTGCAGGCTATT	TTTTTCTTTCGGTTGGTTCA	237	n/a	n/a	n/a
IND4.08329	8.329176	indel	n/a	CTCTTTCTGCGTCTGGTTGG	CGAAGTGGCTGCAATCAAGT	231	n/a	n/a	n/a
IND4.08337	8.337487	indel	n/a	TCTGGTGTTGTAGAGGATGCA	TCAGGCCAACGGTTCACTAT	100	n/a	n/a	n/a
IND4.08348	8.348064	indel	n/a	TTCAAAATCTTTTTGGAAACCA	CCTCTTTATTTTCTCATTTATTGGC	158	n/a	n/a	n/a
IND4.08360	8.360331	indel	n/a	TGAGACAGCATATCACATGGC	CGTGAAAAGTAATGTTTTGTTTGC	237	n/a	n/a	n/a
IND4.08363	8.363944	indel	n/a	TAAGAGCATAAGCAGCGGGT	TTGCTGTGAGGGAAGGAAAC	247	n/a	n/a	n/a
IND4.08370	8.370202	indel	n/a	TGGTGTTTACGTTTCGGTTT	CATACGTTCTCTGTCGGCAA	172	n/a	n/a	n/a
IND4.08378	8.378231	indel	n/a	CGTGGTTCTTTTCGCTCTCC	TCGAACCCAGGAAGAGCTTT	492	n/a	n/a	n/a
IND4.08405	8.405044	indel	n/a	GGACTTTAATCATCTTGCATTCC	AACAATGATTTGGTCCGATTG	271	n/a	n/a	n/a
IND4.08435	8.435557	indel	n/a	GCATACGATCAATTTGCATGTG	AAGTGTTGCAATGCTGGAGG	266	n/a	n/a	n/a
IND4.08510	8.510210	indel	n/a	GAAGTGTCATCGGCGTATACAA	ATGGAATATGGATGTAGAAATTGAT	249	n/a	n/a	n/a
IND4.08708	8.708469	indel	n/a	CGCTTCATTCCACACTCGTA	TGACTCGCTCATTGTCACAT	371	n/a	n/a	n/a
IND4.08893	8.893212	indel	n/a	GATCGATAATGGCAAGCTTCA	TTCCTCGAAGAGCAATGGTT	299	n/a	n/a	n/a
IND4.09009	9.009538	indel	n/a	GGCACTGGCCTTTGAGATAA	TCACCCTTGTTGATGACACG	125	n/a	n/a	n/a
IND4.09096	9.096196	indel	n/a	ACCACCGGAGGCTCTAAAGT	CCCACTCGCCTTGAAAATTA	356	n/a	n/a	n/a
MSAT4.09355	9.355318	microsatellite	n/a	TGGAACAGAGCTTGCATTTG	TGGGGGTTTGTGAAAAAGAG	240	n/a	n/a	n/a
MSAT4.10	9.969057	microsatellite	n/a	GGTAATTTTCTTGGAGACCC	CTAACTAGATCGTCCCTCGT	223	n/a	n/a	n/a
IND4.10019	10.019563	indel	n/a	ATGCCGTCAAACCAAGAATC	GCCATGATTAACGTCGACAA	294	n/a	n/a	n/a
IND4.10161	10.161619	indel	n/a	ATGAAGAACCTCCGATGCTT	TCCCGAACCTTCTCTTTCAG	287	n/a	n/a	n/a
IND4.10237	10.237173	indel	n/a	TTGGAGATTCTTTGGGTTGG	GCTAGGATCCAATGTGATGGA	252	n/a	n/a	n/a
IND4.10256	10.256562	indel	n/a	GGTGCATTCGGAGAAAAGAA	ATTCCTCAAGGCAAAACGTG	227	n/a	n/a	n/a
IND4.10293	10.292719	indel	n/a	AGGCAAATGCCTAATCATGG	ATCGGACGTGCATAGGTTTC	273	n/a	n/a	n/a
IND4.10312	10.312013	indel	n/a	GGGAAGAATCGGGAAACAAT	GTTGCGTTCCAATGGTTCTT	264	n/a	n/a	n/a
IND4.10328	10.328248	indel	n/a	GTCCGGAAAGAGTCATCTCG	AATGGCAACATGGCTACACA	229	n/a	n/a	n/a

IND4.10387	10.387227	indel	n/a	CAAGAAGGCAATATCGGACA	GACGCAATTTGGCTGATTCT	191	n/a	n/a	n/a
IND4.10420	10.420987	indel	n/a	TTCGCTTGGTATGGCTTTTC	CAGCCGCTATGATGTCACAA	229	n/a	n/a	n/a
IND4.10459	10.459982	indel	n/a	TCCGGATCACAAGTTCCAAT	GTTAGGTGGACGATCCGAAA	282	n/a	n/a	n/a
MSAT4.10609	10.609386	microsatellite	n/a	AAATTTGCATCAACCCCAAC	CGGTCTTGCCACTAGCACTT	237	n/a	n/a	n/a
MSAT4.11288	11.290000	microsatellite	n/a	GTCGTCTTTTCATTCGCTGT	TTGGATTTCGATTGCATACC	225	n/a	n/a	n/a
CIW7	11.524362	microsatellite	n/a	AATTTGGAGATTAGCTGGAAT	CCATGTTGATGATAAGCACAA	130	n/a	n/a	n/a
IND4.12333	12.330000	indel	n/a	CGAATTCCTTAAACAATCAAAGC	TCCGGGTTACTGGAGAAGAA	149	n/a	n/a	n/a
IND4.12459	12.459179	indel	n/a	ACACCAATTGGGGATCAAAG	TGAAACTTGTTTGGTTTTGAGG	318	n/a	n/a	n/a
MSAT4.13079	13.079020	microsatellite	n/a	TACTTCGCCACCGTGTGTAA	GTCCGCAACAAACCTTGACT	136	n/a	n/a	n/a

*SNP: Col::Ct
